# A multisite feasibility randomized clinical trial of mindfulness-based resilience training for aggression, stress, and health in law enforcement officers

**DOI:** 10.1186/s12906-024-04452-y

**Published:** 2024-04-04

**Authors:** Michael Christopher, Sarah Bowen, Katie Witkiewitz, Daniel Grupe, Richard Goerling, Matthew Hunsinger, Barry Oken, Tyrus Korecki, Nils Rosenbaum

**Affiliations:** 1 School of Graduate Psychology, Pacific University, 190 SE 8thAve, Suite 260, Hillsboro, OR 97123 USA; 2 Department of Psychology, University of New Mexico, 2001 Redondo S Dr, Albuquerque, NM 87106 USA; 3 Center for Healthy Minds, University of Wisconsin Madison, 625 West Washington Ave, Madison, WI 53703 USA; 4 Department of Neurology, Oregon Center for Complementary and Alternative Medicine in Neurological Disorders, Oregon Health & Science University, 3181 Sam Jackson Park Road, Portland, OR 97239 USA; 5 Department of Psychology, University of California Los Angeles, 1285 Franz Hall, Los Angeles, CA 90095 USA; 6 Behavioral Sciences Department, Albuquerque Police Department, 400 Rome, NW, Albuquerque, NM 87102 USA

**Keywords:** Mindfulness, Resilience, Stress, Law enforcement, Aggression, Remote

## Abstract

**Background:**

Law enforcement officers (LEOs) are exposed to significant stressors that can impact their mental health, increasing risk of posttraumatic stress disorder, burnout, at-risk alcohol use, depression, and suicidality. Compromised LEO health can subsequently lead to aggression and excessive use of force. Mindfulness training is a promising approach for high-stress populations and has been shown to be effective in increasing resilience and improving mental health issues common among LEOs.

**Methods:**

This multi-site, randomized, single-blind clinical feasibility trial was intended to establish optimal protocols and procedures for a *future* full-scale, multi-site trial assessing effects of mindfulness-based resilience training (MBRT) versus an attention control (stress management education [SME]) and a no-intervention control, on physiological, attentional, and psychological indices of stress and mental health. The *current* study was designed to enhance efficiency of recruitment, engagement and retention; optimize assessment, intervention training and outcome measures; and ensure fidelity to intervention protocols. Responsiveness to change over time was examined to identify the most responsive potential proximate and longer-term assessments of targeted outcomes.

**Results:**

We observed high feasibility of recruitment and retention, acceptability of MBRT, fidelity to assessment and intervention protocols, and responsiveness to change for a variety of putative physiological and self-report mechanism and outcome measures.

**Conclusions:**

Results of this multi-site feasibility trial set the stage for a full-scale, multi-site trial testing the efficacy of MBRT on increasing LEO health and resilience, and on decreasing more distal outcomes of aggression and excessive use of force that would have significant downstream benefits for communities they serve.

**Trial registration:**

ClinicalTrials.gov, NCT03784846. Registered on December 24th, 2018.

## Introduction

Policing is a highly stressful occupation [1, 2] that can degrade officers’ mental health, attentional processes, and physiological resilience. Among U.S. law enforcement officers (LEOs), occupational stress has been linked to disproportionately high rates of depression and suicide [3, 4], posttraumatic stress disorder (PTSD) [5], burnout [6], and at-risk alcohol use [7]. Alongside effects on officer health, unmitigated occupational stress also compromises the safety of the communities officers serve, increasing the likelihood of aggression and use of excessive force [8–11]. Although policing is stressful by nature, individual differences in psychological, attentional, and physiological sequalae of stress reactivity play significant moderating roles on the extent to which stressors impact officers’ health and performance.

The impact of stress on LEOs is apparent in elevated rates of mental illness and problematic behavioral patterns. LEOs have high rates of alcohol consumption [12] and binge drinking [13], and death rates due to alcohol-related liver disease among LEOs are twice that of the general population [14]. LEOs who engage in hazardous drinking are four times more likely to report physical violence, and LEOs who meet criteria for an alcohol use disorder are eight times more likely to report intimate partner physical violence [15]. LEO suicidal ideation [16] and suicide rates are up to three times higher than the general public [4], and the number of actual police suicides is likely underreported [17]. LEOs with 15–19 years of experience have the highest rates of completed suicides, which speaks to the cumulative effect of chronic stress exposure on mental health [18].

Attentional and inhibitory processes have also been shown to be negatively affected by stress, which contributes to compromised LEO performance and public safety [19]. Degradation of attentional abilities is related to psychological health, and can increase risk for PTSD, depression, substance use disorder, and suicidality among LEOs [20]. Protecting attentional abilities, such as sustained attention and inhibitory control from stress-related degradation is thus critical in LEO populations.

Neuroendocrine markers play a significant role in physiological reactivity to and recovery from stressors [21, 22]. Prolonged activation of the hypothalamic–pituitary–adrenal (HPA) axis and excessive cortisol release contribute to widespread dysregulation of central and peripheral biological systems influenced by this hormone [23]. Among other deleterious consequences, prolonged HPA axis activation lessens cortisol’s ability to suppress inflammatory responses [24], including C-reactive protein (hs-CRP), an acute phase reactant protein synthesized by the liver, and a triad of inflammatory cytokines (small signaling molecules critical for regulating immune responses), interleukin 6 (IL-6), interleukin 10 (IL-10), and tumor necrosis factor alpha (TNF-⍺). Elevated inflammation, which has been documented in several studies of LEOs [25, 26], is associated with increased aggression [27, 28], PTSD [29], depression [30], and alcohol use disorder [31] and with impaired decision-making [32].

Despite the many risks to LEO health, and the consequential risks to public safety, effective LEO trainings and interventions to mitigate these harms are still lacking. Authors of a meta-analysis [33] of police stress reduction programs concluded that, “insufficient evidence exists to demonstrate the effectiveness of stress management interventions for reducing negative physiological, psychological or behavioral outcomes among police officers.” (p. 508). More recently, in a 2022 meta-analysis of behavioral health interventions [34], authors found collectively these interventions did not reduce stress across LEOs, firefighters, paramedics, and EMTs. Relatedly, LEOs face a number of barriers to seeking mental health services, including stigma and concerns regarding confidentiality, resulting in low use of these services [35, 36].

Interventions suited to the unique context, vulnerabilities, and strengths of this population are needed to improve LEO stress reactivity and psychological health, and to ultimately reduce aggression and violence perpetrated by officers. Mindfulness training has established empirical support in lab-based, clinical, and community-based research, and evidence suggests it is a promising approach for the specific stress-related risks, challenges and outcome patterns present in the LEO population. Mindfulness training has been shown to change how an individual appraises stress, and can increase secondary appraisals of approach-oriented coping resources, thus reducing stress reactivity [37–39]. Recent meta-analyses indicate mindfulness training reduces violent behavior and aggression [40, 41] and improves common LEO health and risk factors, including perceived stress [42–44], depression and suicidal ideation [45, 46], at-risk alcohol use [47, 48], trauma symptoms [49, 50], and burnout [51, 52], and increases psychological resilience [53, 54]. Mindfulness training can also improve sustained attention [55, 56] and inhibitory control [57], and protect cognitive functioning in high-stress cohorts in the context of high-demand periods of work or training [58]. Mindfulness training has also evinced improvement in biomarkers of inflammation and immune system function, including relatively consistent decreases in CRP [59], IL-6, IL-10 [60], TNF-⍺ [61]. Lastly, recent meta-analyses indicate guided mindfulness training delivered online is effective in improving a variety of health outcomes [62, 63], can increase accessibility relative to in-person training [64, 65], and was an effective alternative to in-person training during the peak of the COVID-19 pandemic [66, 67].

Mindfulness training has been shown to be feasible and acceptable, and to improve health outcomes among high-stress populations, such as military personnel and veterans [68, 69], physicians [70, 71], and firefighters [72]. Preliminary evidence supports the feasibility, acceptability, and impact of mindfulness training in reducing psychological and physiological stress reactivity, related negative mental health outcomes, and aggression in LEO samples [73–81]. Despite positive preliminary evidence, these studies generally had small sample sizes, primarily used self-report measures, rarely used randomized controlled trial (RCT) designs (but see [75, 77–79, 81], and did not include an active control group, and only two found sustained benefits at follow-up time points beyond post-intervention [75, 77]. For these reasons, authors of a recent meta-analysis of mindfulness interventions with LEOs [82] encouraged researchers to further explore feasibility and impact of longitudinal RCTs with active control groups in this population.

Due to the preliminary nature and lack of RCTs of mindfulness training with LEO populations, there is a need to identify, optimize, and refine best research practices to inform future efficacy trials assessing effects of mindfulness training among LEOs to ensure generation of valid and reliable results. Therefore, the primary aims of this study were to assess: 1) feasibility and acceptability of recruitment and retention, 2) clinician fidelity to intervention, and 3) responsiveness to change of putative mechanisms and outcome measures including self-report, an attentional task, and markers of peripheral inflammation. The study used a longitudinal, multi-site design in which LEOs were randomized to mindfulness-based resilience training (MBRT), a stress management education (SME) active control, or a no-intervention control (NIC). MBRT was developed with the goal of improving LEO stress reactivity and negative health outcomes associated with officer aggression, thereby potentially providing benefit to officers as well as to the communities they serve.

The current study was not designed as an efficacy trial. As such, no primary or secondary hypotheses were proposed regarding the impact of MBRT and SME on outcomes. Instead, consistent with Consolidated Standards of Reporting Trials (CONSORT) [83] and National Center for Complementary and Integrated Health [84] guidelines for conducting feasibility RCTs, a priori benchmarks for feasibility, acceptability, fidelity, and several indices of responsiveness to change were established to identify optimal primary and secondary outcomes for a future multi-site efficacy trial. These benchmarks are outlined in the Methods section below.

## Methods

### Participants

In collaboration with human resources staff at partnering police departments in three metro areas representing Pacific Northwest, Southwest, and Midwestern U.S. regions, recruitment of sworn LEOs for study participation was conducted via: 1) 10–15-min recruitment sessions led by research staff, 2) email invitations sent to partnering departments, 3) an informational website and flyers posted at department facilities, and 4) community-based police organization leadership. Recruitment included information about the MBRT and SME trainings, randomization, assessed outcomes, concordance between community and investigator goals, and research team contact information. Eligible participants needed to: 1) be 21–65 years old (age limitations for participating police departments); 2) demonstrate English fluency; 3) be a sworn LEO at the rank of Sergeant or below; 4) agree to random assignment to condition; and 5) be willing to complete assessments at multiple time points and attend virtual training groups. Individuals were excluded from participation if they had previously participated in MBRT or a similar mindfulness course (e.g., mindfulness-based stress reduction). Those who successfully met initial criteria were further screened via telephone to assess for ineligibility due to severe depression, suicidal ideation, severe alcohol use, PTSD, or inability or unwillingness to provide informed consent.

### Measures

#### Feasibility and acceptability

Feasibility target indices included several benchmarks: study enrollment (70 participants), acceptance of randomization to condition (≥ 90% acceptance), diversity (across-site average of 20% female and 35% Black, Indigenous, and People of Color (BIPOC) participants), MBRT attendance (≥ 85% of participants attend 6 or more weekly sessions), overall study attrition (≤ 20%), and intervention safety (no study-related adverse or serious adverse events). Acceptability of intervention was assessed in the MBRT and SME groups using four Likert-type scale (1 to 4) items: extent participants liked the training/found it acceptable, likelihood of recommending the course to a fellow officer, likelihood of attending the course again in the future, and reasonableness of assigned home practice (benchmark score of three out of four on all acceptability items, indicating “likely” or “reasonable”). Adherence to assigned homework practice was assessed in MBRT and SME group participants using a smartphone Ecological Momentary Assessment app (mEMA, Ilumivu), downloaded during the first class. The app delivered audio recordings and brief surveys, and tracked time, date, and length of listening for each guided meditation (MBRT) or stress psychoeducation audio-recorded reading (SME).

#### Self-report measures

The Buss-Perry Aggression Questionnaire-Short Form (BPAQ-SF; [85]) is a 12-item scale of aggression derived from the 29-item BPAQ [86]. The BPAQ-SF was developed to assess four dispositional sub-traits of aggression: physical aggression, verbal aggression, anger, and hostility. The BPAQ-SF ranges from 1–5, with higher scores indicating greater aggression. The BPAQ-SF has demonstrated good internal consistency and strong convergent and discriminant validity [87]. The BPAQ-SF demonstrated good internal consistency in the present sample (α_Pre_ = 0.84; α_Post_ = 0.84; α_3-Month Follow-up_ = 0.83; α_6-Month Follow-up_ = 0.81).

PROMIS® (v1.0) short form versions were used to assess sleep disturbance (6 items), alcohol use (7 items), alcohol use negative consequences (7 items), and depression (6 items). PROMIS measures have variable ranges (sleep disturbance [32-76], alcohol use [39-77], alcohol use negative consequences [39-77], anxiety [39-83], and depression [38-80]), with higher scores indicating a higher rate of the measured outcome. Scores were converted to standardized *T* scores (*M* = 50; *SD* = 10), centered on the general U. S. population mean. These short forms have demonstrated acceptable internal consistency and correlations with expected legacy measures [88–90]. In the present sample, internal consistency was in the good to excellent range for alcohol use (α_Pre_ = 0.88; α_Post_ = 0.90; α_3-Month Follow-up_ = 0.91; α_6-Month Follow-up_ = 0.86), alcohol use negative consequences (α_Pre_ = 0.87; α_Post_ = 0.79; α_3-Month Follow-up_ = 0.85; α_6-Month Follow-up_ = 0.87), depression (α_Pre_ = 0.85; α_Post_ = 0.87; α_3-Month Follow-up_ = 0.88; α_6-Month Follow-up_ = 0.88), and sleep disturbance (α_Pre_ = 0.85; α_Post_ = 0.87; α_3-Month Follow-up_ = 0.85; α_6-Month Follow-up_ = 0.88).

Suicidal ideation was assessed using the 7-item Concise Health Risk Tracking scale (CHRT; [91]). The CHRT ranges from 7–35, with higher scores indicating greater suicidal ideation. The CHRT has demonstrated good internal consistency and is correlated with depression and hopelessness [92]. The CHRT demonstrated low to good internal consistency in the present study (α_Pre_ = 0.63; α_Post_ = 0.72; α_3-Month Follow-up_ = 0.85; α_6-Month Follow-up_ = 0.80).

The PTSD Checklist for DSM-5 (PCL-5; [93]) is a 20-item measure based on the DSM-5 symptoms of PTSD. Respondents report how much they were bothered by each symptom over the past month using a 5-point Likert scale ranging from 0–4. Items are summed to create a total score with higher scores indicating greater PTSD symptoms. The PCL-5 has demonstrated good internal consistency and strong convergent and discriminant validity [94]. The PCL-5 demonstrated good internal consistency in the present sample (α_Pre_ = 0.85; α_Post_ = 0.87; α_3-Month Follow-up_ = 0.90; α_6-Month Follow-up_ = 0.91).

The Perceived Stress Scale-10 (PSS-10; [95]) is a 10-item measure used to assess the degree to which situations in life are perceived as stressful. Items are designed to capture how unpredictable, uncontrollable, and overloaded participants find their lives. Items are rated on a 5-point Likert-type scale ranging from 0 to 4, and higher scores indicate greater stress. All items are summed to create a total score. The PSS-10 has shown good internal consistency and demonstrated expected correlations with a variety of constructs [96]. The PSS-10 demonstrated good reliability in the present sample (α_Pre_ = 0.81; α_Post_ = 0.81; α_3-Month Follow-up_ = 0.90; α_6-Month Follow-up_ = 0.85).

The Oldenburg Burnout Inventory (OLBI; [97, 98]) is a 16-item measure of burnout that assesses exhaustion and disengagement from work. The OLBI has acceptable internal consistency, factorial validity, and expected correlations with other constructs [99]. The OLBI ranges from 1–4, with higher scores indicating greater burnout. In the present sample, the OLBI demonstrated adequate internal consistency (α_Pre_ = 0.81; α_Post_ = 0.76; α_3-Month Follow-up_ = 0.76; α_6-Month Follow-up_ = 0.82).

The Five Facet Mindfulness Questionnaire-Short Form (FFMQ-SF; [100]), a 24-item version of the FFMQ [101], assesses dispositional tendency to be mindful in daily life. The observe and describe facets of the scale have demonstrated weaker psychometric properties and issues with novice and non-meditating samples [102, 103]. Thus, the current study used three of the five facets—acting with awareness, nonjudging of experience, and nonreactivity to inner experience. Each facet has five items, resulting in a 15-item scale, with higher scores indicating greater dispositional mindfulness. In the present sample, internal consistency was acceptable to good for nonreactivity (α_Pre_ = 0.81; α_Post_ = 0.80; α_3-Month Follow-up_ = 0.72; α_6-Month Follow-up_ = 0.81), nonjudging (α_Pre_ = 0.75; α_Post_ = 0.71; α_3-Month Follow-up_ = 0.78; α_6-Month Follow-up_ = 0.82); and acting with awareness (α_Pre_ = 0.88; α_Post_ = 0.87; α_3-Month Follow-up_ = 0.88; α_6-Month Follow-up_ = 0.83) facets.

The Self-Compassion Scale-Short Form (SCS-SF; [104]) is a 12-item version of the 26-item SCS [105]. It assesses kindness and understanding toward oneself in instances of pain or failure, perception of one's experiences as part of the larger human experience, and ability to hold painful thoughts and feelings in mindful awareness. The SCS-SF ranges from 12–60, with higher scores indicating greater self-compassion. The SCS-SF demonstrated good internal consistency, factorial validity, and expected correlations with other constructs [104]. The SCS-SF demonstrated good internal consistency in the present sample (α_Pre_ = 0.84; α_Post_ = 0.85; α_3-Month Follow-up_ = 0.85; α_6-Month Follow-up_ = 0.83).

The Brief Resilience Scale (BRS; [106]) is a 6-item measure used to assess psychological resilience. Specifically, the BRS examines the ability to recover or “bounce back” from stressors. A total score is calculated by averaging responses to the 6 items, resulting in a range of 1 to 5 with higher scores indicating greater psychological resilience. The BRS has demonstrated good internal consistency (α = 0.80) and expected correlations with psychological distress, wellbeing [106], and psychological flexibility [81]. The BRS demonstrated good to excellent internal consistency in the present sample (α_Pre_ = 0.82; α_Post_ = 0.83; α_3-Month Follow-up_ = 0.83; α_6-Month Follow-up_ = 0.85).

The Multidimensional Assessment of Interoceptive Awareness-2 (MAIA-2; [107]) is a 37-item measure that assesses interoceptive awareness, or the process by which the nervous system senses, interprets, and integrates signals originating from within the body. Items are rated on a 6-point Likert-type scale ranging from 0–6, and all items are summed to create a total score. Higher scores indicate more adaptive interoceptive awareness. The MAIA-2 has good internal consistency and demonstrated expected correlations with a variety of constructs [107]. The MAIA-2 demonstrated good internal consistency in the present sample (α_Pre_ = 0.89; α_Post_ = 0.80; α_3-Month Follow-up_ = 0.92; α_6-Month Follow-up_ = 0.94).

#### Attentional processes

To assess attention, we utilized the Sustained Attention to Response Task (SART; [108])*.* The SART is a computer-based go/no-go task that assesses sustained attention by measuring a respondent’s ability to withhold a behavioral response to a single, infrequent stimulus presented in the context of repeated responses to frequent stimuli. Participants were first presented with instructions and 178 practice trials. During each practice trial, participants were presented with a digit for 250 ms, followed by a mask for 900 ms. Once the mask disappeared, another digit appeared with no intertrial interval. Participants were instructed to press the space bar when the numbers 1, 2, 4, 5, 6, 7, 8, or 9 appeared (non-targets); they were instructed to provide no response when 3 appeared (targets). Targets appeared on 5% of the trials. Participants were instructed to respond as quickly and as accurately as possible and could respond while the number or mask were on the screen. Trial order was semi-randomized in that target trials were never presented consecutively. After completing practice trials, participants completed 384 critical trials with the same parameters as the practice trials. To measure sustained attention, frequency of two kinds of errors—commission errors (i.e., responses during a target trial) and omission errors (i.e., a lack of response during a non-target trial) were combined, with higher numbers reflecting lower levels of sustained attention.

#### Inflammatory markers

Markers of peripheral inflammation were assessed using dried blood spot (DBS) samples. Specifically, circulating levels of high-sensitivity (hs) CRP and a triad of inflammatory cytokines were assessed: IL-6, IL-10, and TNF- ⍺. Measurement of these biomarkers using DBS is a minimally invasive procedure that allows for blood collection in remote and community-based settings. Instructed by research staff, participants used single-use lancets to prick their own finger and collect 5 drops of blood on filter paper cards, which were subsequently returned to our lab using pre-paid packaging. Samples were stored in the lab in a -20 °C freezer prior to shipping for assay in cold-packed insulated containers. Levels of circulating inflammatory markers were assayed by the lab of Dr. Thom McDade at Northwestern University. hs-CRP was quantified using an updated version of a protocol previously validated for use with DBS samples [109]. IL-6, IL-10, and TNF-⍺ were quantified as described in a recent validation study [110].

#### Procedures

In response to COVID-19-related risks and recommendations, all procedures were conducted remotely via videoconferencing, telephone, and email. A single IRB process was used, and the primary university board approved all study procedures. Interested individuals called the research offices and were read a form asking them to provide verbal consent to complete an initial eligibility screen, which requested contact, employment, and demographic information. Eligible participants provided consent electronically using Qualtrics. Following informed consent administration, participants completed a baseline assessment using a link to a computer-based questionnaire. They were then provided instructions and a separate link to complete a computer-based sustained attention task. Finally, with live instruction from research staff given via Zoom, participants provided a blood sample using a blood spot kit previously sent to them via mail. Upon completion of all baseline assessment procedures, participants were assigned to condition using an individually-randomized group treatment design with a 2:1.5:1 randomization allocation ratio for MBRT, SME, and NIC conditions, respectively. A permuted-block randomization procedure, stratifying by gender, was used to assign participants to study arms.

### Interventions

#### Mindfulness-based resilience training (MBRT)

MBRT is an 8-week training integrating standardized mindfulness practices targeting factors that facilitate stress resilience with cognitive-behavioral treatment and psychoeducation. The general curriculum structure is modeled after the mindfulness-based relapse prevention (MBRP) clinical protocol [111]. Content, context, and language were altered to be more relevant to LEOs, with emphasis on working with reactivity to stressors inherent and unique to police work, including critical incidents, job dissatisfaction, public scrutiny, and interpersonal, affective, and behavioral challenges. Structure and length of sessions was revised to enhance initial “buy-in”, and to better facilitate in-session discussions. For the current trial, MBRT was delivered in 8 weekly live online group sessions via Zoom. The first session was an extended 6-h intensive introduction to mindfulness training, and week 7 was a 4-h intensive practice session. Other sessions lasted 1 h. Sessions contained experiential and didactic exercises including body scan, sitting and walking meditation, mindful movement and discussions. To supplement in-session content and support practice between sessions, participants used the mEMA phone app programmed with audio-guided exercises and monitoring software to track daily playback.

#### Stress management education (SME)

SME, previously designed as an active control condition for other mindfulness-based intervention trials [112], was delivered in 8 weekly live online group sessions via Zoom with extended 4-h sessions on weeks 1 and 4. All other sessions were 2.5 h, such that total session time matched that of MBRT. SME used a group-based didactic approach with modules on physiological and dietary effects of stress, time management, sleep physiology and insomnia, nutrition, exercise, stress hardiness, and factors mitigating impacts of stress. Participants were given weekly homework assignments (amount matched to MBRT). To supplement in-session content, and to match amount and format of assigned homework in the MBRT condition, SME participants also used the mEMA app, programmed with SME-consistent audio content and monitoring software to track daily playback.

MBRT and SME groups were led by separate trainers, with at least masters level training in mental health, exercise or health science, or a related field. MBRT interventionists had previous training in and experience with MBRT or related interventions (i.e., MBRP or MBSR), and underwent intensive training in MBRT delivery, weekly clinical supervision, and regular meetings with the principal investigators to discuss fidelity and other clinical issues. SME interventionists had previous experience leading health education courses, and similarly underwent intensive training, weekly supervision, and regular meetings with expert consultants and principal investigators.

### Fidelity

All MBRT and SME sessions were audio-recorded and coded by independent raters for instructor fidelity. Three of the eight sessions from each cohort were randomly selected using a web-based randomizer, for a total of six coded sessions of each training (SME and MBRT). Separate rating teams coded the two intervention conditions. Each team comprised two doctoral students, not involved in study intervention, who independently rated each of the six randomly selected sessions on adherence to content and instructor skill. Protocol-specified session content was assessed using a 2-point scale (0 = *not present*, 1 = *present*), presence of session themes was assessed using a 4-point scale (0 = *absent*, 1 = *minimally present*, 2 = *present*, 3 = *thorough*), and global ratings of MBRT- or SME-specific skill used a 4-point scale (0 = *none*, 1 = *minimal*, 2 = *adequate*, 3 = *mastery*). The benchmark goal was inter-rater consistency of ≥ 0.75 for coding of group facilitation skills, competence in leading practices and exercises, adherence to session protocols, and overall global rating of the session.

### Data analytic approach

**Fig. 1:**
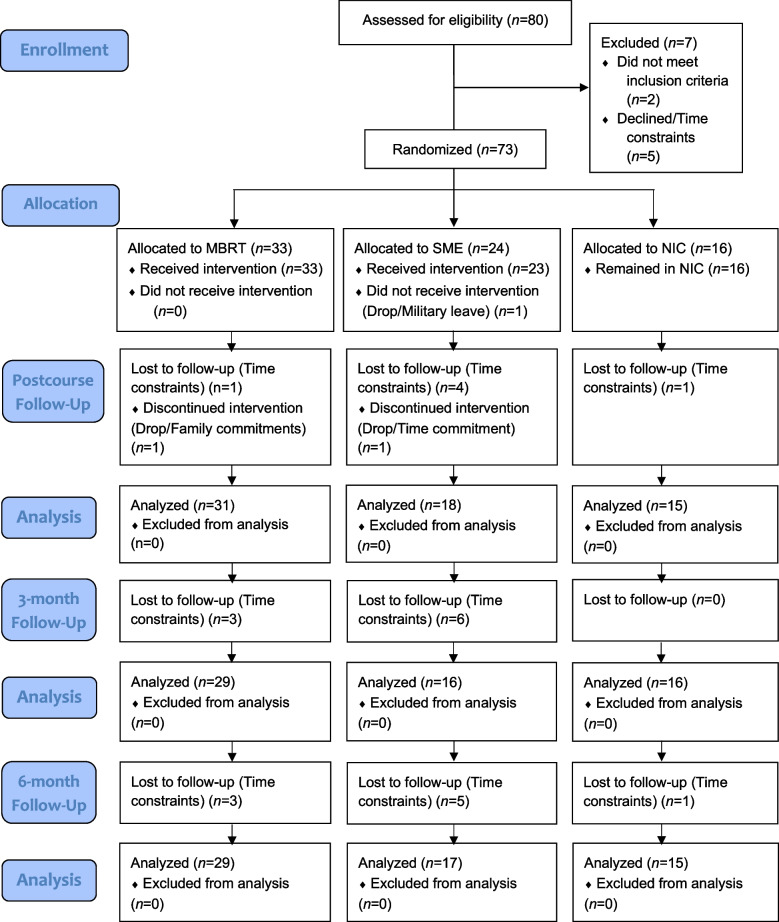
CONSORT participant flow

This study was intended to optimize measurement by assessing the responsiveness to change of conceptually well-justified candidate measures for a future multi-site efficacy trial. Before assessing responsiveness to change, univariate outliers were identified across all outcomes (attentional processes, inflammatory markers, and self-report measures) by calculating the z-score of each outcome at each data collection time point, separately for each arm. For standardized mean response statistics (described below), participants with absolute value z-scores equal to or greater than 3 for the measures for which their scale score was a univariate outlier were filtered out; (2 in NIC; 5 in SME; 7 in MBRT). For relative efficiency analyses and correlations (described below), z-scores were examined, and a square root linear transformation was performed on all scores that contained univariate outliers. Z-scores were then recalculated with transformed scale scores to identify remaining univariate outliers. When outliers were present, participants were filtered out of relative efficiency analyses for measures for which the scale score was a univariate outlier (1 in NIC arm, 3 in SME, 4 in MBRT). For zero-order correlations, this process resulted in no participants being filtered out for the MBRT arm. Participants with a hs-CRP value equal to or greater than 10, thought to reflect a viral or bacterial infection [113], were screened out. However, this screening did not result in the removal of any participants. Prior to conducting analyses, reasons for dropout were examined to assess missingness in the data (see Fig. 1 for CONSORT flow diagram). Results of Little’s Missingness test [114] performed for all time points suggested data were not missing in a systematic fashion (*p’*s > 0.05); complete-case analytic approach was thus employed.

Responsiveness to change was assessed using three methods. First, relative efficiencies were created for each arm using one-way repeated measures ANCOVAs with time as the independent variable (baseline compared to post-training, to 3-month follow-up, and to 6-month follow-up); each outcome (attentional processes, inflammatory markers, and self-report measures) as separate dependent variables; and study site and gender identification as covariates. For each set of analyses, the largest observed *F*-value was divided into the *F*-value for each outcome such that the outcomes with the largest ratios were the most responsive to change from baseline to a subsequent timepoint [115, 116]. Relative efficiency analyses are descriptive and focus on assessing the degree to which measures are sensitive to study arms (MBRT or SME), and not whether change is significant. There are no established benchmarks for these analyses; instead, the goal was to identify which measures were most sensitive to change. Second, the standardized mean response (SMR) was calculated for each arm by dividing the standard deviation of change from baseline to each subsequent timepoint for each outcome into the difference between baseline and each subsequent timepoint [117, 118]. A SMR benchmark of an absolute value ≥ 0.20 (based on the small effect size cut-off for Cohen’s *d* values [119]) in either intervention arm at any follow-up point (i.e., post-training, 3-month follow-up, or 6-month follow-up) was used to identify measures potentially responsive to change. Finally, relationships between changes in putative mechanisms (i.e., inflammatory markers [hs-CRP, IL6, IL10, and TNF-⍺], non-reactivity, and perceived stress) from baseline to post- and 3-month follow-up were examined for the MBRT arm by calculating the correlations between these difference scores and difference scores for aggression (i.e., proposed primary outcome) at corresponding time points [120, 121]. Although the current study was neither a mechanistic nor an efficacy study, consistent with the stress buffering hypothesis [37, 122], the MBRT conceptual model suggests that improvements in physiological (hs-CRP, IL6, IL10, and TNF-⍺) and psychological (perceived stress and non-reactivity) markers are related to reductions in aggression. Difference scores were calculated by subtracting the mean post-training scores from the mean baseline scores and the mean 3-month follow-up scores from the mean baseline scores. Difference scores rather than residualized change scores were used due to the equivalent reliability of both when employing a pure experimental design with no differences across arms at baseline [123]. A benchmark of an absolute value ≥ 0.10 (based on the small effect size cut-off for *r* values [119]) in the MBRT arm at post-training or 3-month follow-up was used to identify potential mechanisms for a fully powered follow-up efficacy trial.

## Results

### Feasibility and acceptability

Feasibility indices were assessed via enrollment, acceptance of randomization, sample diversity, session attendance, study retention, adherence to out-of-session practice, and intervention safety. A final N of 73 participants exceeded the recruitment benchmark of 70, and 95.4% of participants accepting randomization to condition exceeded the acceptance benchmark of 90%. Across three sites, a total of 73 participants were randomized to MBRT (*n* = 33), SME (*n* = 24), or NIC (*n* = 16). The final sample comprised 30.1% female participants and 38.2% BIPOC participants (see Table 1 for participant demographics) exceeding the targets of 20% female and 35% BIPOC participants. Of MBRT participants, 66.7% attended ≥ 6 sessions, and 87.9% attended ≥ 5 sessions, falling short of a goal of 85% of participants attending at least 6 of the 8 sessions. Retention was 87.7% at post-intervention, and 83.6% at both 3-month and 6-month assessment periods, exceeding a goal of 80% retention (see Fig. 1 for CONSORT flow diagram). Lastly, there were no serious adverse events and one non-study-related adverse event.

**Table 1:** Participants sociodemographic characteristics at baseline

	MBRT		SME		NIC			
	*N* or Mean	%	*N or* Mean	%	*N* or Mean	%	*χ*^2^ / *F*	*p*
*N*	33	--	24	--	16	--		
Age (*SD*)	39.57 (10.28)	--	38.08 (6.80)	--	37.06 (10.14)	--	*F* = 0.41	.66
Gender							*χ*^2^ = .127	.93
Female	10	30.3%	7	28%	5	31.2%		
Male	23	69.7%	18	72%	11	68.8%		
Race							*χ*^2^ = 11.67	.07
White	27	81.8%	24	100%	15	93.75%		
Black	0	0%	0	0%	0	0%		
Native Hawaiian/Pacific Islander	0	0%	0	0%	0	0%		
Native American/Alaskan	0	0%	0	0%	0	0%		
Asian	0	0%	0	0%	1	6.25%		
Multi-racial	2	6%	0	0%	0	0%		
Other	4	12.2%	0	0%	0	0%		
Ethnicity							*χ*^2^ = 2.53	.63
Hispanic/Latino	10	30.3%	5	20.8%	4	25%		
Not Hispanic/Latino	22	66.7%	19	79.2%	11	68.8%		
Unknown	1	3%	0	0%	1	6.2%		
Highest Level of Completed Education							*χ*^2^ = 12.96	.53
High School Diploma	1	3%	2	8.3%	0	0%		
1 year of College	1	3%	1	4.2%	2	12.5%		
2 years of College/Associate Degree	7	21.2%	2	8.3%	4	25%		
3 Years of College	4	12.2%	5	20.8%	0	0%		
Bachelor’s Degree	14	42.4%	7	29.2%	6	37.5%		
Bachelor’s Degree plus Graduate Coursework	3	9.1%	2	8.3%	3	18.8%		
Master’s Degree	3	9.1%	4	16.7%	1	6.2%		
Master’s Degree plus Doctoral Coursework	0	0%	1	4.2%	0	0%		
Relationship status							*χ*^2^ = 11.85	.29
Married	24	72.7%	17	70.8%	10	62.4%		
Divorced	0	0%	2	8.3%	1	6.3%		
Widowed	0	0%	0	0%	1	6.3%		
Cohabitating	4	12.1%	1	4.2%	0	0%		
Single	4	12.1%	4	16.7%	4	25%		
Other	1	3.1%	0	0%	0	0%		
Years on the job (*SD*)	11.82 (9.51)	--	10.48 (5.62)	--	9.03 (9.05)	--	*F* = 0.69	.50
Rank							*χ*^2^ = 4.32	.36
Officer	21	63.7%	12	50%	12	75%		
Deputy	2	6%	0	0%	0	0%		
Criminalist	0	0%	0	0%	0	0%		
Detective	4	12.1%	5	20%	1	6.2%		
Sergeant	6	18.2%	6	25%	3	18.8%		
Lieutenant	0	0%	0	0%	0	0%		
Commander	0	0%	0	0%	0	0%		
Captain	0	0%	0	0%	0	0%		
Other	0	0%	1	5%	0	0%		
Serve(d) in the Military (if yes, which branch)							*χ*^2^ = 11.30	.33
Air Force	4	40%	2	25%	0	0%		
Army	3	30%	1	12.5%	2	40%		
Coast Guard	1	10%	0	0%	1	20%		
Navy	0	0%	1	12.5%	0	0%		
Marine Corps	1	10%	3	37.5%	0	0%		
National Guard	1	10%	1	12.5%	2	40%		

*MBRT* Mindfulness-Based Resilience Training, *SME* Stress Management Education, *NIC* no intervention control

Mean responses to 4-point Likert-type scale questions assessing: 1) likability and acceptability of MBRT was *M* = 3.31 (*SD* = 0.93), 2) likelihood of recommending the course to a fellow officer was *M* = 3.59 (*SD* = 0.71), 3) likelihood of attending the course again in the future was *M* = 3.44 (*SD* = 0.62), and 4) reasonableness of assigned home practice was *M* = 3.50 (*SD* = 0.80), all exceeding the minimum benchmark score of 3 on all MBRT items, indicating “likely” or “reasonable”. Mean responses to scale questions assessing: 1) likability and acceptability of SME was *M* = 3.22 (*SD* = 0.58), 2) likelihood of recommending the course to a fellow officer was *M* = 2.90 (*SD* = 0.96), 3) likelihood of attending the course again in the future was *M* = 2.61 (*SD* = 0.98), and 4) reasonableness of assigned home practice was *M* = 3.56 (*SD* = 0.51). Two of four items exceeded the minimum benchmark score of 3 on SME items, indicating “likely” or “reasonable”. When examining engagement with the mEMA app audio mindfulness practices, MBRT participants engaged in an average of 211.66 total minutes of out-of-session practice (*SD* = 186.62; range = 2.12–621.19) over the 8-week training, on an average of 17.11 days out of a possible 56 days (*SD* = 12.69; range = 1–50), with an average of 11.33 min per day on days of practice (*SD* = 3.87; range = 2.12–23.18). SME participants engaged in an average of 65.45 total minutes of out-of-session audiobook listening (*SD* = 68.45; range = 7.03–289.18) over the 8-week training, on an average of 5.25 out of a possible 56 days (*SD* = 5.05; range = 1–22), with an average of 11.12 min per day on days of listening (*SD* = 1.71; range = 7.03–13.58).

### Fidelity

Interventionist fidelity to MBRT and SME protocols was calculated using two-way random-effects model, and percent agreement for items for which there was zero variance in ratings. For coding of MBRT fidelity, inter-rater consistency was *Good* for group facilitation skills (ICC = 0.78), with mean rating indicating *Adequate* to *Thorough* skills (*M* = 3.88; *SD* = 0.34). Raters had 100% agreement on indices of Practices and Exercise Competence and a Global Competence Rating, with the mean rating indicating *Thorough* (*M* = 4; *SD* = 0) and *Thorough/Skillful* (*M* = 4; *SD* = 0), respectively. For indices of adherence, inter-rater consistency was *Substantial*, κ = 0.76, *p* < 0.001 with 96.2% agreement. For SME, inter-rater consistency was *Good* for global rating of instructor skills (ICC = 0.78), and the mean value indicated skill was *Adequate* to *Thorough* (*M* = 3.13; *SD* = 0.34). For indices of adherence, inter-rater consistency was *Moderate*, κ = 0.47, *p* < 0.001 with 82.4% agreement. All indices for MBRT and SME met the benchmark goal of inter-rater consistency of ≥ 0.75.

### Responsiveness to change

Relative efficiencies from baseline to each subsequent time point (i.e., post-training, 3-month, 6-month) for each outcome indicated that responsiveness of measures to change varied across arms. The most responsive measures at post-training were burnout in the MBRT arm, trauma symptoms in the SME arm, and acting with awareness in NIC arm. The most responsive measures at the 3-month follow-up were non-reactivity in the MBRT arm, negative consequences of alcohol use in the SME arm, and depression in the NIC arm. At the 6-month follow-up, the most responsive measures were non-reactivity in the MBRT arm, alcohol use in the SME arm, and aggression in the NIC arm (for a full list of relative efficiencies, see Table 2).

**Table 2:** Responsiveness to change relative efficiency values

Variable	Relative Efficiency (Baseline to post)	Relative Efficiency (Baseline to 3-month follow-up)	Relative Efficiency (Baseline to 6-month follow-up)
hs-CRP			
MBRT	.10	.02	--
SME	< .01	.22	--
NIC	.10	.12	--
IL-6			
MBRT	.02	.16	--
SME	.09	.35	--
NIC	< .01	.10	--
IL10			
MBRT	.09	.50	--
SME	.75	< .01	--
NIC	.12	< .01	--
TNF⍺			
MBRT	.32	.34	--
SME	.06	.59	--
NIC	.46	.10	--
Attention Regulation			
MBRT	.05	.08	.53
SME	.46	.42	.27
NIC	.04	< .01	< .01
Interoceptive Awareness			
MBRT	.02	.12	.03
SME	.34	.24	.08
NIC	.25	.16	.35
Nonjudging			
MBRT	.08	.18	.12
SME	< .01	< .01	.15
NIC	.13	.20	.10
Nonreactivity			
MBRT	.37	1.00	1.00
SME	.05	.05	< .01
NIC	.11	< .01	.03
Acting with Awareness			
MBRT	< .01	.79	.06
SME	.01	.18	.02
NIC	1.00	.07	.36
Self-Compassion			
MBRT	.46	.01	.02
SME	.01	.04	.01
NIC	.22	.03	.55
Resilience			
MBRT	< .01	.58	.60
SME	< .01	.09	.06
NIC	< .01	.05	.36
Alcohol Use			
MBRT	.01	.02	.03
SME	.71	1.00	1.00
NIC	.15	.39	.16
Alcohol Use Negative Consequences			
MBRT	.69	.21	.03
SME	< .01	.01	.50
NIC	.58	.17	.11
Perceived Stress			
MBRT	.01	.06	< .01
SME	< .01	< .01	.18
NIC	.05	.03	< .01
Aggression			
MBRT	.05	.01	.02
SME	.11	.22	.14
NIC	< .01	.01	1.00
Depression			
MBRT	.09	.09	.02
SME	< .01	.03	< .01
NIC	< .01	.01	1.00
Sleep Difficulties			
MBRT	.40	.08	.01
SME	.45	< .01	.26
NIC	< .01	.12	.29
Burnout			
MBRT	1.00	.02	.81
SME	.34	.34	.30
NIC	.22	.37	.48
Suicidal Ideation			
MBRT	.14	.93	.23
SME	.37	< .01	< .01
NIC	.12	.02	.34
Trauma Symptoms			
MBRT	.04	.10	< .01
SME	1.0	.07	< .01
NIC	< .01	.09	.53

*MBRT* Mindfulness-Based Resilience Training, *SME* Stress Management Education, *NIC* no intervention control, *hs-CRP* high sensitivity C-reactive protein, *IL6* interleukin 6, *IL10* interleukin 10 and 10, *TNF⍺* tumor necrosis factor alpha

Examination of standardized mean responses (SMRs) in the MBRT arm revealed a number of standardized change scores ≥|.20| at post, 3-, and 6-month follow. All SMRs with a value of |.20| or greater were in the expected direction over time (suggesting improvement), with the exception of alcohol use at 6-month follow up, which increased. For the SME and NIC arms, there were fewer SMRs with a value of |.20|, and for both groups, and there was a mix of means suggesting some improvement and some worsening of outcomes over time (for a complete list of means and standard deviations at each time point and standardized mean response scores, see Table 3). All measures met the SMR benchmark of an absolute value ≥ 0.20 in either MBRT or SME at either post-training, 3-month follow-up, or 6-month follow-up.

**Table 3:** Responsiveness to change standardized mean responses

Variable	Mean Baseline	Mean Post	Mean 3-month	Mean 6-month	SMR Baseline to post	SMR Baseline to 3-month	SMR Baseline to 6-month
hs-CRP							
MBRT	1.22	.99	.90	--	.26	.35	--
SME	.61	.80	.61	--	-.19	.00	--
NIC	2.11	1.11	1.66	--	1.69	1.50	--
IL6							
MBRT	.55	.58	.37	--	-.06	.35	--
SME	.37	.51	.39	--	-.31	-.07	--
NIC	.28	.23	.23	--	.11	.11	--
IL10							
MBRT	.27	.20	.21	--	.14	.06	--
SME	.28	.17	.18	--	.13	.38	--
NIC	.16	.13	.11	--	.04	.07	--
TNF⍺							
MBRT	2.25	2.02	1.76	--	1.21	2.45	--
SME	2.20	1.71	1.71	--	3.06	2.58	--
NIC	1.87	1.70	1.66	--	.89	1.50	--
Attention Regulation							
MBRT	17.03	15.86	15.33	15.88	.10	.19	.07
SME	16.00	13.50	9.30	9.53	.35	.67	.60
NIC	31.42	30.71	25.35	29.23	.03	.13	.05
Interoceptive Awareness							
MBRT	3.88	4.24	3.98	4.19	-.63	-.47	-.53
SME	3.91	3.92	4.08	4.06	-.02	-.38	-.29
NIC	3.65	3.94	3.73	3.72	-1.07	-.21	-.23
Nonjudging							
MBRT	3.50	3.50	3.68	3.68	.00	-.25	-.26
SME	3.48	3.60	3.42	3.33	-.23	.09	.25
NIC	3.74	3.60	3.67	3.84	.24	.12	-.16
Nonreactivity							
MBRT	3.40	3.61	3.55	3.60	-.28	-.19	-.29
SME	3.78	3.78	3.60	3.70	.00	.23	.10
NIC	3.48	3.53	3.68	3.44	-.05	-.24	.04
Acting with Awareness							
MBRT	3.54	3.55	3.55	3.58	-.02	-.01	-.06
SME	3.52	3.60	3.54	3.46	-.18	-.04	.09
NIC	3.81	3.96	3.87	3.83	-.34	-.18	-.03
Self-Compassion							
MBRT	3.34	3.52	3.60	3.65	-.42	-.47	-.55
SME	3.63	3.64	3.60	3.51	-.02	.06	.27
NIC	3.22	3.49	3.50	3.46	-.75	-.93	-.60
Resilience							
MBRT	3.71	4.08	3.98	4.11	-.56	-.59	-.74
SME	4.00	4.16	4.11	4.11	-.23	-.15	-.17
NIC	3.96	4.02	4.02	3.57	-.23	-.30	1.15
Alcohol Use							
MBRT	46.61	46.95	48.00	47.82	-.06	-.20	-.23
SME	45.10	46.85	47.70	44.43	-.27	-.39	.30
NIC	45.52	46.94	46.43	50.57	-.30	-.12	-.68
Alcohol Use Negative Consequences							
MBRT	40.67	40.98	40.80	41.25	-.06	-.02	-.11
SME	40.33	40.79	41.54	40.28	-.14	-.29	.04
NIC	40.49	41.37	42.00	41.59	-.25	-.22	-.25
Perceived Stress							
MBRT	24.45	23.38	22.65	22.96	.22	.41	.21
SME	22.57	21.93	23.42	23.30	.09	-.11	-.10
NIC	21.50	21.30	22.07	22.38	.04	-.11	-.24
Aggression							
MBRT	1.93	1.71	1.62	1.57	.55	.70	.67
SME	1.59	1.59	1.56	1.61	.00	.09	-.07
NIC	1.67	1.74	1.69	1.58	-.25	-.04	.26
Depression							
MBRT	50.14	49.21	49.50	48.98	.15	.11	.13
SME	47.41	46.78	48.78	48.16	.06	-.15	-.08
NIC	47.60	46.43	46.85	44.78	.20	.19	.47
Sleep Difficulties							
MBRT	54.35	52.02	51.45	51.35	.33	.41	.47
SME	52.37	54.47	52.81	49.52	-.23	-.06	.39
NIC	52.82	51.76	52.41	51.30	.27	.08	.39
Burnout							
MBRT	2.40	2.39	2.34	2.32	.03	.21	.18
SME	2.38	2.31	2.33	2.39	.26	.14	-.03
NIC	2.35	2.29	2.34	2.35	.32	.04	.00
Suicidal Ideation							
MBRT	8.84	8.74	8.53	7.92	.05	.13	.45
SME	7.63	7.66	8.00	7.92	-.02	-.24	-.11
NIC	7.50	8.53	8.07	8.00	-.68	-.29	-.23
Trauma Symptoms							
MBRT	35.16	32.10	32.96	30.64	.33	.25	.52
SME	32.10	29.60	32.42	32.07	.36	-.02	.00
NIC	29.71	29.69	30.78	29.23	.00	-.22	.09

*SMR* standardized mean response, *MBRT* Mindfulness-Based Resilience Training, *SME* Stress Management Education, *NIC* no intervention control, *hs-CRP* high sensitivity C-reactive protein, *IL6* interleukin 6, *IL10* interleukin 10 and 10, *TNF⍺* tumor necrosis factor alpha

When examining relationships between changes in putative mechanisms (i.e., inflammatory markers [hs-CRP, IL6, IL10, and TNF-⍺], non-reactivity, and perceived stress) and changes in aggression at post-training in MBRT, correlations between changes in aggression and IL6 (*r* = 0.36), IL 10 (*r* = 0.12), TNF-⍺ (*r* = 0.36), perceived stress (*r* = 0.24), hs-CRP (*r* = 0.07), and non-reactivity (*r* = -0.32) were in the expected direction and in line with the conceptual model predicting a decrease in aggression as stress and stress-reactivity markers improve. At 3-month follow-up, all correlations were in the expected direction: *r*_IL6_ = 0.34, *r*_hs-CRP_ = 0.14, *r*_IL10_ = 0.25, *r*_TNF_⍺ = 0.28, *r*_Perceived stress_ = 0.46, and *r*_Non-reactivity_ = -0.19, and met the minimum benchmark of an absolute value ≥ 0.10 at 3-month follow-up.

## Discussion

The current study was a longitudinal, multi-site randomized 3-arm feasibility trial in a sample of LEOs in three diverse geographical regions of the U.S. The study assessed feasibility and acceptability of recruitment and retention, clinician fidelity to intervention, and responsiveness to change of putative mechanisms and outcome measures including self-report assessments, an attentional task, and physiological inflammation markers. The ultimate goal of this work was to inform future efficacy trials assessing effects of mindfulness training on psychological, attentional, and physiological outcomes among LEOs.

Results generally supported feasibility and acceptability benchmarks for recruitment and retention. The number of participants recruited and the acceptance rate of randomization to condition both exceeded the associated goals. The goal for recruitment of a diverse sample was also met, for both female participants and BIPOC participants. Despite estimates that only 13% of sworn LEOs in the U.S. are female [124], several previous trials enrolled high percentages of female LEOs (e.g., 36% [73] U.S., 40% [75] US, 53% [74] US, 56% [76] Netherlands, and 75% [77] Brazil). Consistent with a wide variety of mindfulness training trials, women may be more likely to participate in mindfulness research [125], even in an historically predominantly male profession. The rate of BIPOC LEOs in this study exceeded previous trials in the U.S., which averaged approximately 15% BIPOC [73–75, 126]. Including racially marginalized people in mindfulness research, and LEOs trials in particular, is essential given promising health and societal impacts [127].

In the current study, 87.9% and 66.7% and of participants attended at least 5 or 6 sessions, respectively, comparable to studies in which 88% attended 5 or more sessions [76] and 69% attended 6 or more sessions [77], but short of the approximately 85% attendance rate in other recent studies [74, 75]. However, in those trials, participants attended intervention sessions while “on the clock” which may have boosted attendance. With changes in work shifts and occurrence of unanticipated work-related duties, it may not be feasible to expect 85% of LEOs to attend 6 or more sessions. Alternative formats for the course may thus be worth exploring, such as an immersion-based delivery of MBRT with the same overall intervention time delivered over 2 days followed by remotely-delivered brief “booster” sessions. Preliminary data suggests this model is feasible and results in similar outcomes as an 8-week model [128].

At the 6-month follow-up, retention (83.6%) exceeded our benchmark (≤ 20% attrition). Attrition rates in previous mindfulness training trials with LEOs have been variable, ranging from 7% attrition at 5-month follow up [74] to up to 41% at the end of the 8-week intervention [78]. In a recent meta-analysis [129], authors identified a 19% attrition rate (operationalized as loss to follow-up at post-test) across 114 mindfulness training RCTs, which is slightly above the 16% attrition in this study.

Finally, regarding intervention acceptability, the benchmark was met with a majority of MBRT participants indicating that they liked the training/found it acceptable, would recommend it to a fellow officer, would attend the course again in the future, and found the amount of assigned home meditation practice reasonable. Although participants found SME to somewhat be less acceptable, the benchmark was met for two key items (i.e., likability and reasonability of home practice, and just below cutoff for likelihood of recommending the training to a fellow officer), suggesting that SME is a tolerable active control condition. Similar to many mindfulness trainings, participants were asked in MBRT to integrate practices learned in sessions into their daily lives. The current study assessed between-session practice using an app that recorded time spent engaged with the provided audio recorded practices. Leveraging newer mobile technologies to more accurately assess intervention engagement and enactment is an exciting direction in recent and for future research. The current study audio recorded intervention sessions and trained independent coders to assess fidelity using a detailed coding system. Benchmarks for inter-rater consistency and for interventionist adherence and competence were also met.

Responsiveness to change was assessed in three ways – relative efficiencies, standardized mean responses (SMRs), and correlations. Analyses focused on the relative differences among observed *F*-values, SMRs, and correlation coefficients. The quantitative goal was to identify measures most responsive to change in relation to the set of measures in the data set. When examining relative efficiencies, inconsistent results were identified across arms. In the MBRT arm, burnout and non-reactivity overlapped to some degree across post-baseline time points. This is not surprising given MBRT’s focus on cultivating non-reactivity. These results suggest that these two constructs are important ones to measure in future trials. In SME, the measure of alcohol use was one of the more efficient measures. Examination of means across time points reveal that problematic alcohol use actually numerically increased from baseline to the post-training and 3-month follow-up time points; if this numerical increase is statistically meaningful, then the measure was efficient in detecting change across time, suggesting it may be an important measure for future trials. In the NIC group, measures of depression and burnout emerged as relatively more efficient measures, suggesting these measures may be useful to include in future trials. Even though relative efficiencies in the SME and NIC arms provide valuable information, information from the MBRT arm is relatively more valuable. If a construct did not change over time in the SME or NIC arm, then the relative efficiency value would be close to zero, which would reflect responsiveness to change. Given that it is not known which constructs were likely to change in the SME and NIC arms simply due to time, there is ambiguity in the meaning of a small relative efficiency value in these arms.

When examining change without taking in account any covariates with the SMRs, measures of aggression and interoceptive awareness emerged as relatively more responsive measures, not surprising given targets of MBRT, and may be important measures in future trials. Moreover, the measure of interoceptive awareness was relatively more responsive to numerical change across all arms. Interestingly, across all arms of the study, TNF-⍺ emerged as a responsive measure with SMRs reflecting numerical improvement, suggesting inclusion of inflammatory markers in future trials. All measures met the SMR benchmark of an absolute value greater than or equal to 0.20 in either MBRT or SME at post-training, 3-month follow-up, or 6-month follow-up.

Lastly, when examining correlation coefficients in MBRT, changes in inflammatory markers, stress, and non-reactivity showed consistent relationships with aggression at 3-month follow-up. The pattern of correlations was consistent with the conceptual model for the impact of MBRT on aggression, and met the minimum benchmark of an absolute *r* value greater than or equal to 0.10, suggesting that improvement in inflammatory markers, stress, and non-reactivity may be important to include as putative mechanisms in future MBRT trials. Collectively, these results indicate the self-report, attentional, and inflammatory markers included in this study are responsive to change and warrant inclusion in a follow-up multisite efficacy trial.

Alongside evidence of feasibility, acceptability, and responsiveness to change outcomes, there are limitations that warrant caution in interpretation of results. First, the study was designed to assess multi-site feasibility and acceptability, and was not designed to assess efficacy; therefore, the sample size does not support direct comparisons of change over time across the three conditions. Second, similar to military samples, police samples may be prone to underreporting mental health symptoms due to stigma and concerns regarding confidentiality [130]; therefore, the mean endorsed values for several outcomes, such as suicidal ideation and alcohol use, may be lower than actually experienced. Third, although attentional control and physiological indices were included in the study design, many other outcomes were self-report, presenting a further potential limitation. Relatedly, although aggression and non-reactivity were assessed, on-the-job performance measures would enhance the scope of assessment. Because departments participating in the study used different methods to track officer infractions, use of force, and other relevant occurrences, it was not possible to combine these data for assessment. Future multi-site trials will require careful planning to ensure equivalence of indices across departments and regions. Fourth, although the attrition rate met the a priori benchmark, future research with LEOs and other groups of first responders should explore various delivery schedules, such as briefer session lengths, intense immersion models, and integrating the training into the workplace. Fifth, although police stressors were assessed, other factors such as non-work-related stress and level of social support were not, which may have limited the scope of our assessment.

Despite these limitations, the current study adds to a burgeoning literature suggesting mindfulness training may mitigate effects of unique occupational stressors on law enforcement. In addition to demonstrating feasibility and acceptability, preliminary data suggest MBRT may lead to improvement in aspects of LEO psychological health and risk, aggression, and stress reactivity. Future trials should focus on supporting enactment of regular mindfulness practice following course completion, address barriers to practice, and perhaps provide “booster” sessions to support training gains. While exposure to trauma and stressors is inherent in policing, programs that train officers to relate to these experiences more skillfully may help reduce the harmful effects of stress on their own health as well as their interactions with the broader community.

## Data Availability

The datasets in the current study are available from the corresponding author on reasonable request.
